# Effectiveness of an mHealth system on access to eye health services in Kenya: a cluster-randomised controlled trial

**DOI:** 10.1016/S2589-7500(21)00083-2

**Published:** 2021-06-21

**Authors:** Hillary Rono, Andrew Bastawrous, David Macleod, Ronald Mamboleo, Cosmas Bunywera, Emmanuel Wanjala, Stephen Gichuhi, Matthew J Burton

**Affiliations:** a International Centre for Eye Health, London School of Hygiene & Tropical Medicine, London, UK; b Kitale County Referral and Teaching Hospital, Kitale, Kenya; c Peek Vision, Berkhamsted, Hertfordshire, UK; d MRC Tropical Epidemiology Group, London School of Hygiene & Tropical Medicine, London, UK; e Department of Ophthalmology, Kenyatta National Hospital, University of Nairobi, Nairobi, Kenya; f Moorfields Eye Hospital NHS Trust, London, UK

## Abstract

**Background:**

There is limited access to eye health services in many low-income and middle-income populations. We aimed to assess the effectiveness in increasing service utilisation of the Peek Community Eye Health (Peek CEH) system, a smartphone-based referral system comprising decision support algorithms (Peek Community Screening app), SMS reminders, and real-time reporting.

**Methods:**

In this cluster-randomised controlled trial of eye health in Kenya, community unit clusters were defined as one health centre and its catchment population. Clusters were randomly allocated (1:1) to receive Peek CEH and referral (intervention group) or standard care via periodic health centre-based outreach clinics and onward referral (control group). Individuals in the intervention group were assessed at home by screeners and those referred were asked to present for triage assessment in a central location. They received regular SMS reminders. In both groups, community sensitisation was done followed by a triage clinic at the cluster health centre 4 weeks after sensitisation. During triage, individuals in both groups were assessed and treated and, if necessary, referred to a specific hospital. Individuals in the intervention group received further SMS reminders. The primary outcome was the mean attendance rate (the number of people per 10 000 population) at triage of those with confirmed eye conditions, as assessed at 4 weeks after sensitisation in the intention-to-treat population. We estimated the intervention effect using a Student's t-test on cluster-level rates. This trial is registered with Pan African Clinical Trial Registry, number 201807329096632.

**Findings:**

Between Nov 26, 2018, and June 7, 2019, of the 85 community units in Trans Nzoia County, Kenya, 49 were excluded. We randomly allocated 18 community units each to the intervention group (68 348 individuals) and the control group (60 243 individuals). 9387 individuals from the intervention group and 3070 from the control group attended triage assessment. The mean attendance rate at triage by individuals with eye problems was 1429 (92% CI 1228–1629) in the intervention group and 522 (418–625) in the control group (rate difference 906 per 10 000 [95% CI 689–1124; p<0·0001]).

**Interpretation:**

The Peek CEH system increased primary care attendance by people with eye problems compared with standard approaches, indicating the potential of this mobile health package to increase service uptake and guide appropriate task sharing.

**Funding:**

The Queen Elizabeth Diamond Jubilee Trust and Wellcome Trust.

## Introduction

Globally, 596 million people have distance vision impairment, 43 million of whom are blind.^1^ Most people with vision impairment live in low-income and middle-income countries (LMICs).^1^ Many factors contribute to high prevalence of vision impairment in LMICs, including poverty, lack of awareness, scarce health-care professionals specialised in eye health, and concentration of health-care services in major urban areas.^2^ Additionally, in-demand secondary services are often utilised by people with minor conditions that could be managed in primary care.^3^ The 2019 World Report on Vision calls for the advancement of eye health through integration into Universal Health Coverage, with an emphasis on strengthened services within primary health care.^4^ Primary care for eye-related ailments is delivered using different approaches, including via primary health-care staff trained in basic eye care delivered at fixed community-based facilities or via occasional outreach clinics by a specialist eye care team to primary health-care facilities.^5^ This second approach is currently used in Kenya.

The past two decades have witnessed increased mobile phone coverage, affordability, and capability in Kenya.^6^ There are several mobile health (mHealth) interventions seeking to improve communication with individuals, promote access to health services, provide a clinical diagnosis, encourage treatment adherence, and support management of chronic diseases.^7^ A recent meta-analysis of mHealth interventions reported that mHealth interventions are more effective at improving health outcomes than comparators.^8^ Although there are several publicly available health apps worldwide, only a small number have been evaluated in randomised controlled trials.^9^

**Research in context**

**Evidence before this study**

In sub-Saharan Africa, eye care provision is insufficient to meet the minimum requirements set out by WHO in the World Report on Vision, leading to many people becoming or remaining visually impaired or at risk of becoming blind from treatable or preventable eye conditions. Poor access to and awareness of eye services are key barriers, arising in part from the presence of very few eye care providers in the health system for the levels of unmet need. One of the strategies to improve access to eye care is the redistribution of tasks among health workforce teams, to improve efficiency across the available human resources. A systematic review on the effectiveness of mobile health (mHealth) interventions that support communication between health-care providers and individuals through SMS appointment reminders showed evidence of benefit in the provision of health care. At the start of this trial, we searched PubMed, Global Health, and Google Scholar for relevant literature published between Jan 1, 2000, and Dec 31, 2017, using the search terms “mHealth”, ”eye health”, and “Africa”. Our search had no language restrictions. We selected articles that were peer-reviewed primary research studies of any study design, studies that measured the effect or uptake of an mHealth intervention in eye care, studies done in an African setting, and studies published from 2000 onwards (period during which mHealth interventions have been in use). We did not find any publications, thus there was no evidence from trials using mhealth in eye care, but there were some mHealth interventions focused on managing HIV, tuberculosis, and malaria. During the conduct of this trial, a few randomised trials were published on the topic of mHealth interventions in eye care, mainly from outside Africa.

**Added value of this study**

To our knowledge, this is the first randomised controlled trial using mHealth to support the uptake of eye services in sub-Saharan Africa. Our trial provides evidence that integration of technology into primary health systems could increase access to eye care services, increase adherence to referrals, and support more appropriate utilisation of services. Our results show that individuals who were not trained in eye health could use the Peek Community Eye Health system to identify and refer people with eye problems accurately. This mHealth intervention also integrated into the community, primary, and secondary services; improved access to care, adherence to referrals, and equity of access; and facilitated more appropriate use of the required level of services compared with the current standard of care.

**Implications of all the available evidence**

Our results indicate the potential of this technology to improve uptake of eye services, enable sex and age equity in eye health, and guide task shifting of case identification to help target scarce resources. Furthermore, establishing primary eye care services and integrating them into community-based activities and secondary care services leads to increased management of primary eye conditions at primary care facilities, thereby leaving the secondary facilities to deal with more complex and sight threatening cases rather than overburdening them with unnecessary referrals that can be managed in primary care. The learnings from this trial have been adopted by the Ministries of Health in Kenya to inform a national scale-up programme.

Peek Acuity is a validated smartphone app that reliably identifies people with vision impairment who need eye care services.^10^ We previously did a cluster-randomised controlled trial in Kenya to detect vision impairment in school children by teachers using Peek Acuity.^11^ Teachers could reliably screen for vision impairment. The uptake of referrals to hospital in the intervention group was more than twice that of the control group, suggesting mHealth solutions can improve access to eye health services.

Before starting this trial, we developed and validated the Peek Community Screening application (PCS app). The PCS app combines a decision algorithm and Peek Acuity, which enables more accurate referral decisions by community volunteers.^12^ We then integrated this app into an mHealth system for eye health screening in communities.

In this study, we aimed to test the hypothesis that this integrated Peek Community Eye Health (Peek CEH) system increases access to eye services through increased identification of people with eye problems in the community; increases uptake of a referral within 4 weeks by those with an identified eye problem; and results in more appropriate utilisation of primary and secondary care services in the community.

## Methods

### Study design and participants

We did a single-masked, parallel-group, cluster-randomised controlled trial of the Peek CEH system in 36 clusters, called community units, in Trans Nzoia County, Kenya. The study setting was a rural area and no other eye health interventions were occurring at the time of the trial. Our mHealth intervention was compared with the current standard of care (periodic health centre-based outreach clinics and referral). Community units linked to private or secondary health facilities were excluded.

In the intervention group, all households within each community unit were visited and individuals had their visual acuity tested using the PCS app. All individuals with reduced visual acuity or reporting any eye problem were referred to the linked primary health-care facility for assessment on a specified date by an outreach team. Those with conditions requiring secondary level assessment and treatment, such as cataract or refractive error, were referred to hospital for secondary eye care at Kitale Eye Unit.

In the control group, residents with self-reported eye problems were invited to attend a periodic eye health triage clinic, which was held on a specified date at the local primary health-care facility and was operated by an outreach team from Kitale Eye Unit. Individuals in both groups attending this service were assessed, treated, and, when necessary, referred. Referred individuals in both groups received a written hospital referral letter, those in the intervention group also received SMS reminders.

Each community unit was defined as one dispensary or primary health-care centre together with the population they serve. In Kenya, a typical community unit has 5000 to 10 000 people.^13^ To avoid confounding, none of the randomised trial community units bordered one another. We did a baseline census in all community units before the trial to determine the population size per cluster. All people present in the community unit during the study were eligible for inclusion. Written informed consent from all participants was obtained by a trained field research team before enrolment; parents or guardians consented for children. Participants were excluded from the study if they did not provide consent. Residence, age, and sex were recorded at enrolment.

The study was approved by the Moi University Institutional Research and Ethics Committee (Nairobi, Kenya) and the London School of Hygiene & Tropical Medicine Ethics Committee (London, UK). Permission was also granted by the Department of Health (Trans Nzoia County, Kenya). The study adhered to the principles of the Declaration of Helsinki on Ethics. The trial protocol is published elsewhere.^14^ This study follows CONSORT guidelines for reporting cluster randomised trials.^15^

### Randomisation and masking

36 community units (18 per group) were randomly assigned to either the Peek CEH system (intervention group) or standard care (control group). Restricted randomisation was used to ensure balance between the groups for: distance from Kitale Eye Unit, direction from Kitale Eye Unit (north, south, east, and west quadrants), and subcounty. The randomisation restriction rules have been described previously.^14^ Briefly, a set of 10 000 allocations of community units to groups, that met the restriction criteria, were computer generated by a statistician who did not participate in recruitment, and one of these permutations was randomly selected. Neither the study participants nor the field research team could be masked to group allocation; however, the statistician, hospital registration clerk, and clinicians assessing outcomes were unaware of group allocation.

### Procedures

For both groups, we sent posters and verbal notices to churches and schools 4 weeks before the outreach clinic date, encouraging people with eye problems to self-report to the clinic for an eye check-up.

In the intervention group, eye health was actively screened at the household level. From a pool of community volunteers, we selected 18 Peek screeners and trained them to use the PCS app. Peek screeners were selected on the basis of their ability to use a smartphone and travel to multiple communities. A local community volunteer from the community guided the screener. The screener and the local community volunteer visited each household. They were reimbursed for transport and meal expenses, and were supervised by trained field research team members. The screeners used the app to identify people with eye problems. The test algorithm prompts screening questions, such as: “Do you have any discomfort or pain in your eyes today?” and “Do you have a problem with your sight when seeing far or near objects?”. Parents or guardians of children were asked “Does the child have any problem with their eyes today?”. The app then prompts the testing of distant visual acuity for individuals aged 6 years and older. Additionally, if an individual is aged 40 years or older, the app prompts near vision assessment using the RADNER reading chart at 40 cm, with all results recorded in the app.^16^ Distance visual acuity of each eye was tested separately, and near vision tested binocularly.^10^ We recorded age and sex for each individual. Individuals who were absent during house visits were asked to join the examination team at the next household or on the next day at another location within the same cluster. An individual screened positive if they had visual acuity of less than 6/12 (20/40) in either eye; had any self-reported eye pain or discomfort; self-reported difficulty seeing distant or near objects; or were not able to see N8 on near vision assessment. After a positive screening result, the app prompted the collection of additional information for contact and follow-up purposes: name, guardians or parent's name if a child, primary language, and contact telephone number, and generated a referral SMS notification to the linked primary health-care facility. The app sent these referral details to a cloud-based server, which automatically generated a personalised SMS to the individual with referral information (date and location). Weekly reminder messages were sent, the last one being 1 day before the appointment date. Individuals were also given a referral letter.

In the control group, there was no active screening of vision in the community. Individuals with symptomatic eye problems were alerted to services through community sensitisation and invited to attend local primary health-care facilities on a pre-advertised date. On that day, the Kitale Eye Unit team held an outreach clinic. When referral was needed, individuals were given an identical referral letter to the ones used in the intervention group. They did not receive SMS reminders. Each letter had a unique code number to link the patient referral record to their Kitale Eye Unit attendance.

For both groups, on the day of triage clinic, a team from Kitale Eye Unit visited the community unit's primary health-care facility. Data on clinic attendance were entered into a dedicated cloud-based system, generating unique identifier numbers. Individuals were assessed using the current standard outreach procedures and equipment (Snellen chart for testing visual acuity, magnifying loop, refraction, and direct ophthalmoscopy) and were either treated on site or referred to Kitale Eye Unit for further assessment. Eye health interventions provided in the outreach clinic included reading spectacles, eye drops, and removal of foreign bodies. When referred to Kitale Eye Unit, a pre-numbered referral letter with their study number, name, date, reason for referral, and triage centre was provided to individuals of both trial groups. Upon arrival, triage attendees were asked for their address. If they had come from outside the community unit, they still received the same treatment as attendees from the community unit; however, they were excluded from any analyses to avoid potential confounding of the effect estimates.

For the intervention group, immediately after referral from the primary health care, an SMS was sent to the participant or guardian asking them to present to Kitale Eye Unit. For up to 4 weeks after the primary health care assessment, weekly reminder SMS were sent to those who had not yet attended their Kitale Eye Unit referral. On arrival at Kitale Eye Unit, individuals presented their referral letter, which was identical between groups to maintain masking, and a clerk recorded the attendance of the referred individual.

The clinical team assessed individuals to identify ocular problems and the treatment needed. Visual acuity was measured using a 6-metre Snellen chart. vision impairment was defined as <6/12 in either eye. Diagnoses were classified using International Classification of Diseases 10.^17^ Distance between the primary health-care facility and Kitale Eye Unit was estimated using Google maps.^18^ All participants received free treatment at Kitale Eye Unit, regardless of trial group.

### Outcomes

The primary outcome was the proportion of the population attending the triage clinic at the community unit's local primary health-care facility with a confirmed eye condition (true positive) determined by the visiting hospital team, at 4 weeks from the time of initial sensitisation. We refer to this as the attendance rate, expressed as the number of attendees per 10 000 population.

The secondary outcomes were the proportion of the population who attended the triage clinic without any eye condition (false positives) and proportion of the population who attended Kitale Eye Unit after a referral from the triage clinic. We also report, as a secondary outcome, the proportion of participants who attended Kitale Eye Unit within 4 weeks among only those who were referred at triage, and the time taken by individuals to attend. The diagnoses made at triage and Kitale Eye Unit by trained ophthalmic clinical officers were used to determine the appropriateness of referrals. Primary and secondary outcome data were collected by a hospital registration clerk who was unaware of participant-level group assignment.

### Statistical analysis

To determine the sample size of 36 clusters we used the Hayes formula for rates in unmatched cluster-randomised trials.^19^ In Trans Nzoia County, a typical health facility has a catchment population of 5000 people.^13^ During previous community outreach programmes to these health facilities, about 50–100 new individuals attended.^20^ This estimate translates to an average of 150 new individuals with eye problems per 10 000 population (about 75 people with eye problems per community unit). We estimated that the community units would vary from 75 to 225 individuals with eye problems, giving an estimated coefficient of variation (k) of 0·25, and, therefore, an intraclass correlation coefficient of 0·001. To achieve a desired power of 90% at a significance level of 5%, a sample of 36 community units (18 in each group) would be sufficient to detect a difference of 0·5%, from 1·5% in the control group to 2·0% in the intervention group (a 33% relative change) in overall attendance rates from the community to the primary care facility at 4 weeks (the primary outcome).

All outcomes were analysed in the intention-to-treat population and the primary analysis was done at cluster level. We calculated the proportion of individuals attending triage within each cluster, by dividing the number attending triage with a confirmed eye condition by the cluster population, determined at baseline census. We did Student's t-tests on cluster-level proportions to provide an estimate of attendance rate difference (with 95% CIs) between the two groups, and we used the p value to assess the strength of evidence against the null hypothesis that the rate does not differ between the two groups.^21^ We tested whether this effect was modified by sex, age, or distance from Kitale Eye Unit. For distance, since it was a cluster-level covariate, we did a linear regression analysis on the cluster level attendance rates and included distance and trial group as exposures with an interaction term between them. For age and sex, we used the approach recommended by Cheung et al for testing for effect modification of individual-level covariates in a cluster-level analysis.^22^ Where evidence of effect modification was seen, stratum specific estimates are presented. The proportion of the population attending triage clinic who were not found to have an eye condition (false positive attendance rate) was estimated in a similar manner in both groups.

We also estimated the effect of the intervention on the attendance rate of residents arriving at Kitale Eye Unit via a referral from primary health-care triage, using the same approach as the cluster-level analysis of the primary outcome. The numerator of each community unit was the number of individuals attending Kitale Eye Unit after a referral from triage and the denominator was the community unit population.

Among those referred to Kitale Eye Unit, we tested whether there was a difference in the odds of attending Kitale Eye Unit within 28 days of referral between groups using a logistic regression model, adjusted for age, sex, distance to Kitale Eye Unit, and community unit included as a random effect. We also investigated whether time-to-attendance at Kitale Eye Unit post-referral was different between the groups. First, we checked visually using Kaplan-Meier plots then tested formally using Cox regression, adjusted for age, sex, and distance from Kitale Eye Unit, and community unit as a random effect.

We tabulated data on individuals' visual acuity and diagnosis of eye problems at both triage and Kitale Eye Unit. We used STATA version 15 (STATA Corp, College Station, TX, USA) for all analyses.^23^ This trial is registered with Pan African Clinical Trial Registry, number 201807329096632.

### Role of the funding source

The funders of the study had no role in study design, data collection, data analysis, data interpretation, or writing of the report.

## Results

**Figure 1:**
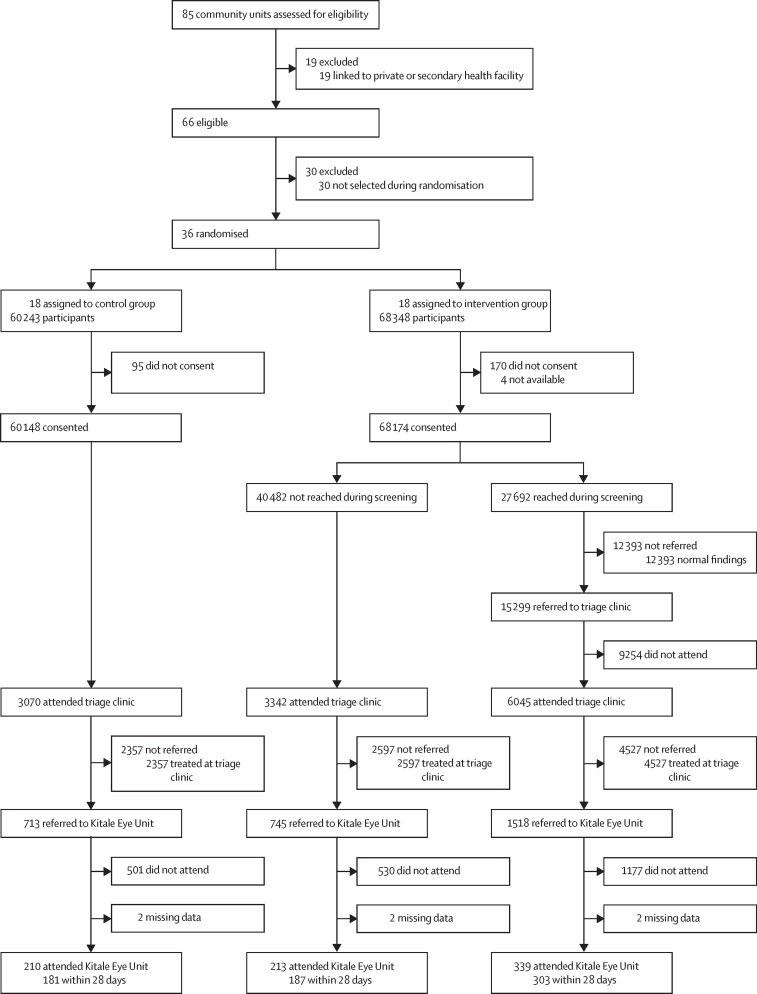
Trial profile

Between Nov 26, 2018, and June 7, 2019, in Trans Nzoia County, Kenya, of the 85 potentially eligible community units, 19 were excluded as they were linked to private or secondary heath facilities. Of the remaining 66 eligible community units, 30 were excluded and 18 were randomly allocated to each group (figure 1).

We enumerated 128 591 people in 36 community units; 68 348 (53·2%) lived in the 18 intervention group community units (table 1). The intervention clusters tended to be larger than the control clusters, with a mean cluster population of 3797 (SD 1397) for the intervention and 3347 (955) for the control. The mean distance from the community units to Kitale Eye Unit was 18·3 km (SD 9·9) in the intervention group compared with 19·8 km (7·9) in the control group (appendix p 1). Overall, 50·6% of those enumerated were female and 44·4% were younger than 15 years, with age and sex balanced between study groups (table 1). Consent to participate was granted by 128 322 (99·8%) of 128 591 people. Those who did not consent were not screened, but were welcome to attend the triage clinic if they had any eye problems; therefore, we used the enumerated population as the denominator for estimating the attendance rate.

**Table 1:** Demographic characteristics of eligible participants

		**Control group**			**Intervention group**		
		Enumerated*	Attended triage†	Attended hospital†	Enumerated*	Attended triage†	Attended hospital†
All participants		60 243	3070 (5·1%)	210 (0·4%)	68 348	9387 (13·7%)	552 (0·8%)
Age group, years							
	<15	26 755 (44·4%)	743 (2·8%)	26 (0·1%)	30 317 (44·4%)	1843 (6·1%)	56 (0·2%)
	15–29	15 705 (26·1%)	445 (2·8%)	38 (0·2%)	18 102 (26·5%)	1203 (6·6%)	98 (0·5%)
	30–44	8604 (14·3%)	473 (5·5%)	34 (0·4%)	9387 (14·8%)	1640 (17·5%)	97 (1·0%)
	45–59	5066 (8·4%)	730 (14·4%)	47 (0·9%)	5561 (8·1%)	2360 (42·4%)	116 (2·1%)
	60–74	2629 (4·4%)	456 (17·3%)	46 (1·7%)	3162 (4·6%)	1677 (53·0%)	120 (3·8%)
	≥75	785 (1·3%)	223 (28·4%)	19 (2·4%)	975 (1·4%)	664 (68·1%)	65 (6·7%)
Mean age, years (SD)		22·4 (18·5)	38·9 (24·1)	44·8 (23·1)	22·4 (18·7)	41·6 (23·2)	46·1 (23·0)
Sex							
	Female	30 405 (50·5%)	1759 (5·8%)	107 (0·4%)	34 629 (50·7%)	5706 (16·5%)	301 (0·9%)
	Male	29 838 (49·5%)	1311 (4·4%)	103 (0·3%)	33 719 (49·3%)	3681 (10·9%)	251 (0·7%)

Data are n (%) unless otherwise stated.

* Denominator is all enumerated within the trial group.

† Denominator is those enumerated within the category of age or sex in the trial group.

In the intervention group, 68 174 (99·7%) individuals consented; of these, 27 692 (40·6%) were screened. The proportion of individuals screened varied across community units (range 21·8–92·4%), with mean community unit coverage of 42·8% (SD 18·5). The main reason individuals were not screened was that they were absent during the screening visit. Older individuals were more likely to be screened, with about 99% of those aged 75 years and older and 90% of those aged 60–74 years being screened; for those younger than 15 years, screening reduced down to only 20%.

Of the 27 692 individuals screened at home, 15 299 (55·2%), screened positive for an eye problem and were referred to the triage clinic; of whom, 6045 (39·5%) attended the triage clinic in the primary health-care facility (figure 1). A further 3342 individuals from the intervention community units attended triage without having received a referral, and 3070 individuals attended triage in the control community units, which gave 12 457 individuals attending the community unit triage clinics. 11 862 (95·2%) of these 12 457 individuals were diagnosed by a clinician as having some form of eye problem and were either treated or referred to Kitale Eye Unit. Overall, 9·2% of the total population in the 36 trial community units (as enumerated) attended triage and had a clinician-confirmed eye problem.

The mean attendance rate at the triage clinic of those with confirmed eye problems (the primary outcome) in the control group was 522 per 10 000 residents (95% CI 418–625) and 1429 per 10 000 (1228–1629) in the intervention group. This provided strong evidence (p<0·0001) that the rate of attendance was higher in the intervention group than the control group, with an estimated difference of 906 attendees per 10 000 residents (95% CI 689–1124; table 2). There was some variation between community units in the attendance rate, which ranged from 831 to 2091 per 10 000 individuals in the intervention group and 80 to 901 per 10 000 in the control group. Of the 19 community units with the highest attendance rates, 18 were intervention community units. The observed coefficient of variation (k) was 0·40 in the control group and 0·28 in the intervention group, equivalent to intraclass correlation coefficients of 0·009 and 0·013 respectively. These findings suggest that there was higher variability within the clusters in both the control and intervention groups; the clusters allocated to either the control or intervention were not homogeneous.

**Table 2:** Estimated mean attendance rates for the community unit triage clinic

		**Control group**	**Intervention group**	**Difference**	p value*	**p**_interaction_**value**†
Overall		522 (418–625)	1429 (1228–1629)	906 (689–1124)	<0·0001	NA
Sex						
	Female	586 (463–709)	1712 (1462–1961)	1125 (858–1393)	<0·0001	<0·0001
	Male	455 (362–549)	1137 (982–1292)	682 (507–856)	<0·0001	..
Age group, years						
	<15	270 (212–328)	610 (482–737)	340 (205–475)	<0·0001	<0·0001
	15–29	262 (206–319)	652 (532–772)	389 (262–517)	<0·0001	..
	30–44	550 (427–673)	1761 (1498–2024)	1211 (931–1490)	<0·0001	..
	45–59	1459 (1171–1746)	4423 (3840–5005)	2964 (2338–3590)	<0·0001	..
	60–74	1839 (1348–2330)	5762 (4662–6862)	3923 (2763–5083)	<0·0001	..
	≥75	2987 (2278–3696)	7429 (5872–8986)	4442 (2794–6089)	<0·0001	..

Data are rates per 10 000 (95% CI) unless otherwise stated. NA=not applicable.

* From Student's t-tests for differences in attendance rates.

† From Student's t-tests of the differences seen in each category. For age, interaction tested between those younger than 45 and 45 and older.^22^

**Figure 2:**
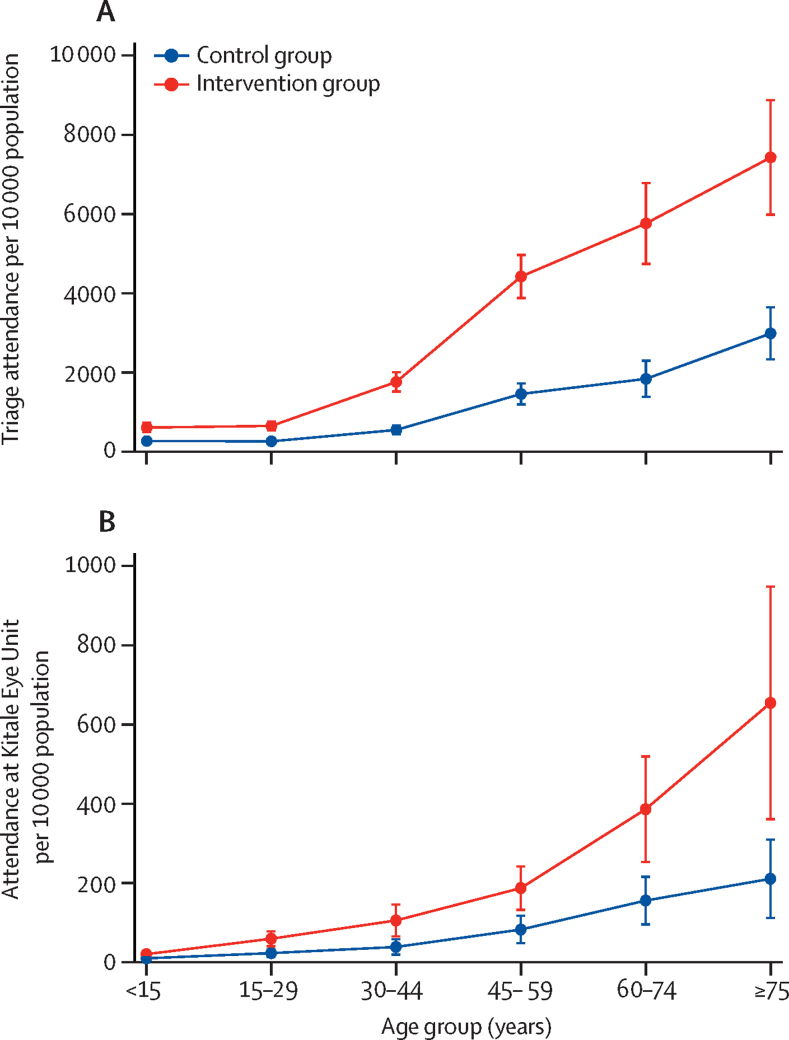
Mean attendance rate among individuals who attended triage (A) and the hospital at Kitale Eye Unit within 28 days (B)

The intervention effect differed between male and female participants (p_interaction_<0·0001) and differed by age (p_interaction_<0·0001). The increased attendance rate at the triage clinic in the intervention group, compared with the control group, was greatest in females and older individuals (table 2, figure 2A). There was no evidence of effect modification by distance between community unit and Kitale Eye Unit (p=0·89).

Of the 12 457 individuals who presented for triage, 594 (4·8%) had no eye problems identified by the study clinician (false positives) and there was no evidence of a difference between study groups (4·7% for intervention and 5·0% for control; odds ratio [OR] 0·94, [95% CI 0·78–1·14]; p=0·52). However, the estimated mean rate of attendance of people without any eye problems at triage clinics among the total community unit population was higher in the intervention group (72 per 10 000 residents [95% CI 51–92]) than in the control group (26 per 10 000 residents [95% CI 17–34]), giving an estimated difference of 46 per 10 000 residents (95% CI 25–67; p=0·0001).

2976 individuals were referred to Kitale Eye Unit after attending a community unit triage clinic, of whom six were missing data related to hospital attendance (figure 1). Of the 2970 individuals with follow-up data, 762 (25·7%) attended Kitale Eye Unit, with 671 (22·6%) arriving within 28 days. The median time to hospital attendance was 16 days (IQR 9–22) with a mean of 17·9 days (SD 13·5).

The estimated number from the community unit population (as enumerated) referred via the community unit triage clinics and attending Kitale Eye Unit (within 28 days of referral) was estimated to be 33 per 10 000 residents (95% CI 23–43) in the control group and 82 per 10 000 (59–105) in the intervention group (table 3); a difference of 49 attendees per 10 000 residents (95% CI 25–73; p=0·0002). As with the primary outcome, there was evidence that the intervention effect differed by age and sex (table 3, figure 2B).

**Table 3:** Estimated mean attendance rates for the Kitale Eye Unit

		**Control group**	**Intervention group**	**Difference**	p value*	**p**_interaction_**value**†
Overall		33 (23–43)	82 (59–105)	49 (25–73)	0·0002	NA
Sex						
	Female	32 (23–41)	90 (64–116)	59 (32–86)	0·0001	0·028
	Male	35 (21–49)	73 (52–94)	39 (15–63)	0·003	..
Age group, years						
	<15	9 (5–13)	20 (11–29)	11 (1–20)	0·029	0·0008
	15–29	22 (12–33)	59 (39–79)	36 (14–58)	0·002	..
	30–44	38 (17–59)	105 (62–148)	67 (21–113)	0·006	..
	45–59	82 (45–119)	187 (127–246)	105 (37–172)	0·003	..
	60–74	155 (90–220)	386 (242–529)	231 (79–382)	0·004	..
	≥75	210 (104–316)	654 (338–970)	444 (123–765)	0·008	..

Data are rates per 10 000 (95% CI) unless otherwise stated. NA=not applicable.

* From Student's t-tests for differences in attendance rates.

† For age, interaction was tested between those younger than 45 years and those aged 45 years and older.^22^

Of the 711 individuals in the control group with follow-up data who were referred to Kitale Eye Unit, 181 (25·5%) presented to the hospital within 28 days compared with 490 (21·7%) of 2259 in the intervention group. After adjusting for community unit clustering, distance from the hospital, whether they were categorised as visually impaired at the triage clinic, age, and sex, the evidence of a between-group difference in attending hospital within 28 days was weak (p=0·145), with the observed odds of attendance lower in the intervention group than in the control group (adjusted OR 0·77 [95% CI 0·54–1·10]; table 4). Use of a multivariable logistic regression model showed that hospital attendance within 28 days was most likely among men, those younger than 75 years, people with vision impairment, and those living closer to Kitale Eye Unit (table 4).

**Table 4:** Participants who attended Kitale Eye Unit within 28 days of being referred from triage clinic

		**Referred**	**n (%)**	Adjusted odds ratio*	**95% CI**	**p value**
Control group		711	181 (25·5%)	1 (ref)	..	..
Intervention group		2259	490 (21·7%)	0·77	0·54–1·09	0·145
Age group, years						
	<15	298	74 (24·8%)	1 (ref)	..	..
	15–29	514	124 (24·1%)	1·03	0·73–1·45	0·860
	30–44	494	122 (24·7%)	1·06	0·75–1·50	0·732
	45–59	665	143 (21·5%)	0·82	0·59–1·14	0·246
	60–74	592	139 (23·5%)	0·89	0·63–1·25	0·492
	≥75	407	69 (17·0%)	0·56	0·38–0·82	0·003
Sex						
	Female	1763	361 (20·5%)	1 (ref)	..	..
	Male	1207	310 (25·7%)	1·40	1·17–1·68	0·0003
Visual status at triage						
	6/12 (20/40) or better in both eyes	1085	227 (20·9%)	1 (ref)	..	..
	Worse than 6/12 (20/40) in either eye	1885	444 (23·6%)	1·29	1·06–1·58	0·011
Distance to hospital, km						
	≤10	448	124 (27·7%)	1 (ref)	..	..
	11–20	1574	369 (23·4%)	0·84	0·53–1·36	0·482
	>20	948	178 (18·8%)	0·56	0·34–0·92	0·022

Odds ratio, 95% CI, and p values are derived from a multivariable logistic regression model, adjusted for cluster using a random effects model.

* Adjusted for clustering, trial arm, age group, sex, visual status at triage and distance to hospital.

**Figure 3:**
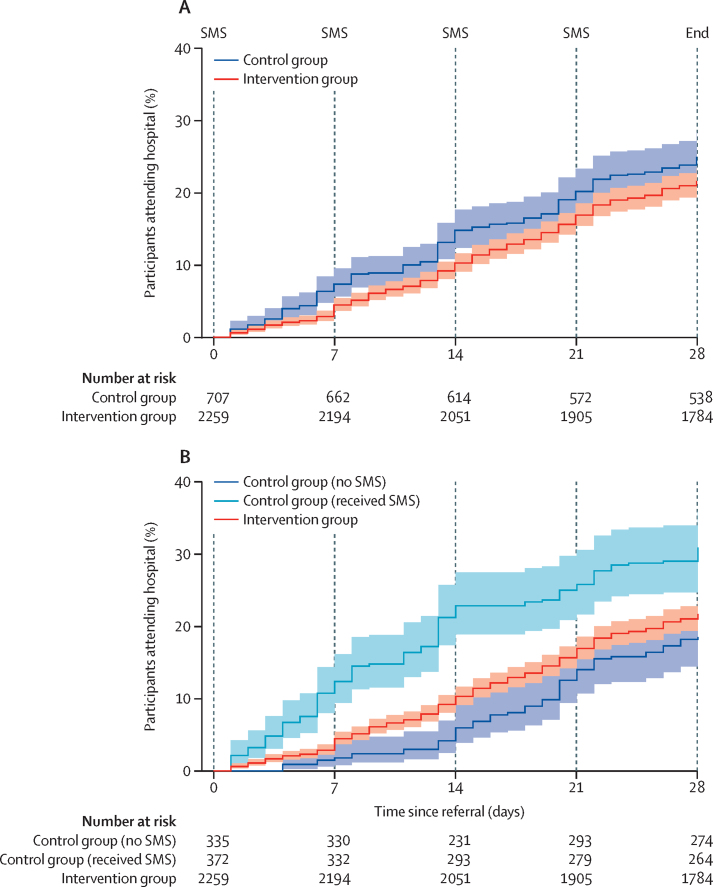
Kaplan-Meier analysis of time from triage referral to attendance at the hospital at Kitale Eye Unit, among all individuals (A) and stratified by those who erroneously received an SMS (B) With adjustment for age, sex, and distance from Kitale Eye Unit, p values from Cox regression for the association between study group and time to hospital attendance were p=0·145 (A) and p=0·015 (B).

Individuals in the intervention group took slightly longer on average to attend Kitale Eye Unit after referral than did those in the control group (figure 3A); however, the evidence for a true difference in the time-to-attendance was weak, with an adjusted hazard ratio of 0·80 (95% CI 0·59–1·08; p=0·14).

The conditions diagnosed in those presenting at the community unit triage clinic are listed in the appendix (p 2). 8492 (68·2%) of 12 457 individuals had allergic or other forms of conjunctivitis, presbyopia or no eye conditions that could appropriately be managed at primary health care level. Of the remaining conditions, the most common were cataracts (10·9%), refractive error excluding presbyopia (10·6%), and retinal diseases (2·3%). Overall, 9481 (76·1%) individuals who attended the community unit triage clinic were treated in that location.

762 individuals attended Kitale Eye Unit; however, 43 of these individuals checked in at the hospital reception but left before being clinically assessed. Of the 719 assessed, 62·9% had either cataracts, refractive error, retinal diseases, or glaucoma, conditions that were appropriate for the hospital, but the rest (17·1%) had allergic conjunctivitis (10·3%), conjunctival growths (5·7%), or normal findings (1·1%) that could have been managed earlier (appendix p 2).

In error, nine of 18 control clusters received SMS reminder messages that should only have been delivered to the intervention group after referral from triage. Therefore, we repeated the analyses involving hospital attendance, separating out the clusters into three groups—control group (no SMS), control group (received SMS), and intervention group (received SMS)—to assess the effect, if any, of contamination on the intervention. Comparison of attendance at 28 days across the three groups showed a difference in attendance rates (p=0·058 for comparison between three ORs). After adjustment for clustering in the community, age, sex, vision status, and distance, estimated attendance in the intervention group was similar (OR 1·04 [95% CI 0·66–1·63], p=0·87) to that of the control group (no SMS); estimated attendance was higher than in the control group (received SMS) than in the intervention group (OR 1·62 [1·08–2·41], p=0·018). However, attendance in the control group (received SMS) was estimated to be higher (OR 1·68 [1·00–2·83], p=0·050) than in the control group (no SMS). This finding is consistent with the time-to-attendance analysis, with an estimated hazard ratio of 1·11 (95% CI 0·75–1·65, p=0·59) when comparing the intervention group with the control group (no SMS), and a hazard ratio of 1·72 (95% CI 1·10–2·69, p=0·017) when comparing the control group (no SMS) with the control group (received SMS; figure 3B).

## Discussion

The Peek CEH system increased attendance at primary health-care and secondary level facilities, while simultaneously improving the appropriateness of where services were delivered to the given population.

Utilisation of eye care services in the control group was comparable to another Kenyan study that found a 4·8% response.^24^ Our trial showed that a combined system comprising a smartphone-based decision support algorithm to identify eye problems (PCS app) integrated into the Peek CEH system improved the overall attendance rate among people with eye problems, increasing access. This finding is likely to be due to identification of people with eye problems and improved communication and reminders on accessing services.

Health systems need to improve the targeting of people to the right services. Previously, we reported a high proportion (61%) of people accessing secondary care eye services could have been managed in the primary health-care setting, potentially displacing people who need more specialist services.^3^ In this trial, the reverse pattern was found in the intervention group, with most minor conditions being managed in the primary health-care setting and most of those attending a secondary service having more complex problems. Although we do not have more recent data on hospital utilisation, we found that hospital utilisation (secondary care) by the catchment population during the study (2018–19) remained consistent with annual levels seen in 2013–15 (80 per 10 000);^3^ however, a major improvement in the proportion of appropriate utilisation of secondary eye services in comparison with 2013–15 was seen, with 17·1% (previously 61%) having primary eye care conditions (mostly managed at triage) and 62·9% (previously 8%) having priority vision impairing eye conditions.

WHO advocates for a well-coordinated eye care system with each level of health care having specific roles, such as management of cataracts and refractive errors in secondary care, and basic eye care provision in primary health care.^4^ Our findings suggest it might be possible to shift management of some eye conditions to primary health care, supported by specialist eye health practitioners, which would increase capacity at secondary level for more complex conditions.

One limitation in previous studies of smartphone vision impairment detection was false positives.^11^ In our study, there was no difference in false positives between groups (4·7% for intervention *vs* 5·0% for control), although the absolute number of people without eye problems was higher in the intervention group than in the control group due to an overall increase in the number of people accessing services. Although the benefits of the intervention to the population outweigh this increased, unnecessary demand on services, there is potential for overburdening the national health system. The risk might be reduced by providing triage services close to the communities to review those who screened positive and to manage minor eye ailments and, where capacity allows, the assessment and delivery of presbyopia correction, which accounted for 26% of individuals seen at triage. Human resource capacity for eye health in primary care in LMICs in general needs to be assessed and strengthened before scale-up. Our findings showed that non-specialists using smartphone decision support algorithms can reliably identify and refer individuals with eye problems, enabling them to share the burden of this task.

Our mHealth intervention increased utilisation of eye services by the population both in primary care (triage and basic treatment) and secondary care (hospital), more so in women than men and across all ages, although there were possibly barriers among participants who needed secondary care. Previous studies found that secondary services were less utilised by young people and older women,^3^ which suggests that our intervention could improve equity, especially among women and those aged 45 years or older. Improvement in uptake could be due to increased awareness of and engagement with services.^25^

There was a barrier between home and accessing primary eye care in the community. There was also reduced hospital attendance from the intervention group (even though evidence of a difference is weak), suggesting the possibility of a SMS threshold above which further SMS intervention has no additional effect. The individuals might have lost trust in the health system if they did not receive the service when they were first told to attend; a qualitative study is needed to answer this question. Factors associated with reduced hospital attendance included: female sex, long distances to the health facilities, older age groups, and those without any visual impairment (despite having a confirmed eye problem). Other barriers that were not assessed include poverty, costs, or fear of treatment.^26^ Previous findings suggest that the influence of sex depends on the level of health care being accessed. When services are closer, access by women is higher than more distant services; however, when accessing secondary services, other considerations might arise. These considerations include prevailing social norms where men have authority in family decisions; for example, in some communities women needed to seek permission from their husbands before going to hospital.^27^ It could also be due to other competing priorities deemed of greater importance than seeking eye care, such as childcare or other economic activities (eg, farming in the rainy season).

We found access to secondary care in those with vision impairment was more probable than in those without vision impairment. This is possibly because of a greater perceived need or impact of the condition on their functioning. We found higher utilisation among older people, probably because vision impairment increases with age.^28^ However, among people 75 years and older, utilisation was lower than in younger age groups, probably indicating barriers to accessing care, such as traditional beliefs, acceptance of vision impairment as part of aging, or lack of support to access services.^29^

This trial has a number of strengths. We did a full enumeration to accurately define the study population. The trial was integrated into the existing health system, informing adoption and scale-up.^30^ It represents the first community cluster randomised controlled trial of an mHealth intervention to increase access to eye care services in sub-Saharan Africa. The primary outcome measure of attendance was objective and robust.

This trial has limitations. There was an intervention error in the control group that involved participants in nine of the 18 clusters receiving SMS reminders. When we did stratified multivariate analyses, more individuals in the control group (received SMS) attended their hospital appointment and came faster than those in the control group that received no SMS and the intervention group. This finding suggests that perhaps the novelty of the SMS reminders wears off over time or that multiple SMS reminders might have caused some intervention fatigue; therefore, a carefully determined balance is needed on the frequency of SMS reminders that would create the required awareness. There was low screening coverage, suggesting other screening approaches need to be explored. A process evaluation was not done; however, the results from an economic evaluation are being analysed.

This trial has provided evidence that integration of community, primary, and secondary health services using the Peek CEH system might reduce the burden of eye disease and vision loss by simultaneously increasing access to vital services for the population by ensuring individuals are seen at the appropriate level of the health system while also potentially increasing service capacity and productivity. The learnings from this trial have been adopted by the Ministries of Health in Kenya to inform a national scale-up of eye care screening programme. The benefits of using the Peek CEH system outweigh the risks of unnecessary referrals.

## Data sharing

In line with the requirements of the ethics committees that approved this research, requests for access to data should be made in writing to the corresponding author. De-identified participant data can be made available, along with a data dictionary, to researchers who obtain ethical approval for their proposed analysis and provide a signed data-sharing contract, which enables data storage and analysis for a time-limited period.

## Declaration of interests

MJB is a Trustee of The Peek Vision Foundation. AB is CEO of The Peek Vision Foundation and Peek Vision Ltd and receives salary support from Peek Vision. HR and CB are consultants employed by Peek Vision Ltd. All other authors declare no competing interests.
