# Supplementary material for: Effectiveness of an mHealth system on access to eye health services in Kenya: a cluster-randomised controlled trial
Source: Lancet Digit Health. 2021 Jun 21;3(7):e414–24. doi: 10.1016/S2589-7500(21)00083-2 (PMC8239618; doi:10.1016/S2589-7500(21)00083-2)
Supplement: Supplementary appendix [file mmc1.pdf]

THE LANCET
Digital Health

Supplementary appendix

This appendix formed part of the original submission and has been peer reviewed. We post it as supplied by the authors.

Supplement to: Rono H, Bastawrous A, Macleod D, et al. Effectiveness of an mHealth system on access to eye health services in Kenya: a cluster-randomised controlled trial. *Lancet Digit Health* 2021; **3:** e414–24.

Table S1: cluster characteristics

		Control arm	*Intervention arm*
Distance from KEU	Mean (SD)	19.8km (7.9)	18.3km (9.9)
Population	Mean (SD)	3347 (955)	3797 (1397)
Number of clusters per Subcounty	Cherangani	7	7
	Endebes	2	2
	Kiminini	2	3
	Kwanza	4	4
	Saboti	3	2
Number of clusters in each direction from KEU	East	5	5
	South	5	5
	West	3	4
	North	5	4

KEU – Kitale Eye unit

Table S2: Diagnosis of the participants who attended Triage and Hospital appointments at Kitale Eye Unit in Trans Nzoia county, Kenya.

Eye conditions	Triage				Hospital*			
	Control arm		Intervention Arm		Control arm		Intervention Arm	
	n/3070	%	n/9387	%	n/199	%	n/520	%
Allergic conjunctivitis	841	(27·4%)	2518	(26·8%)	21	(10·6%)	53	(10·2%)
Presbyopia	718	(23·4%)	2609	(27·8%)	9	(4·5%)	22	(4·2%)
Other Conjunctivitis	355	(11·6%)	857	(9·1%)	1	(0·5%)	10	(1·9%)
Cataracts	324	(10·6%)	1038	(11·1%)	60	(30·2%)	128	(24·6%)
Refractive errors	320	(10·4%)	1006	(10·7%)	50	(25·1%)	153	(29·4%)
Normal	153	(5·0%)	441	(4·7%)	2	(1·0%)	6	(1·2%)
Retinal diseases	92	(3·0%)	199	(2·1%)	14	(7·0%)	39	(7·5%)
Conjunctival growths	60	(2·0%)	162	(1·7%)	13	(6·5%)	28	(5·4%)
Corneal diseases	53	(1·7%)	127	(1·4%)	4	(2·0%)	11	(2·1%)
Glaucoma	31	(1·O%)	47	(0·5%)	1	(0·5%)	7	(1·4%)
Eye injury & FB in eye	11	(0·4%)	29	(0·3%)	2	(1·0%)	3	(0·6%)
Chalazion & lid swellings	7	(0·2%)	35	(0·4%)	4	(2·0%)	14	(2·7%)
Uveitis	4	(0·1%)	20	(0·2%)	2	(1·0%)	3	(0·6%)
Lid inflammations	4	(0·1%)	14	(0·2%)	0	(0·0%)	14	(2·7%)
Others	97	(3·2%)	285	(3·0%)	16	(8·0%)	43	(8·3%)

* 43 participants checked into the hospital but left before being attended to.
